# Supplementary material for: Aptamer-Targeted Plasmonic Photothermal Therapy of Cancer
Source: Mol Ther Nucleic Acids. 2017 Aug 16;9:12–21. doi: 10.1016/j.omtn.2017.08.007 (PMC5582647; doi:10.1016/j.omtn.2017.08.007)
Supplement: Document S1. Figure S1 [file mmc1.pdf]

OMTN, Volume *9*

Supplemental Information

Aptamer-Targeted Plasmonic

Photothermal Therapy of Cancer

Olga S. Kolovskaya, Tatiana N. Zamay, Irina V. Belyanina, Elena Karlova, Irina Garanzha, Aleksandr S. Aleksandrovsky, Andrey Kirichenko, Anna V. Dubynina, Alexey E. Sokolov, Galina S. Zamay, Yury E. Glazyrin, Sergey Zamay, Tatiana Ivanchenko, Natalia Chanchikova, Nikolay Tokarev, Nikolay Shepelevich, Anastasia Ozerskaya, Evgeniy Badrin, Kirill Belugin, Simon Belkin, Vladimir Zabluda, Ana Gargaun, Maxim V. Berezovski, and Anna S. Kichkailo

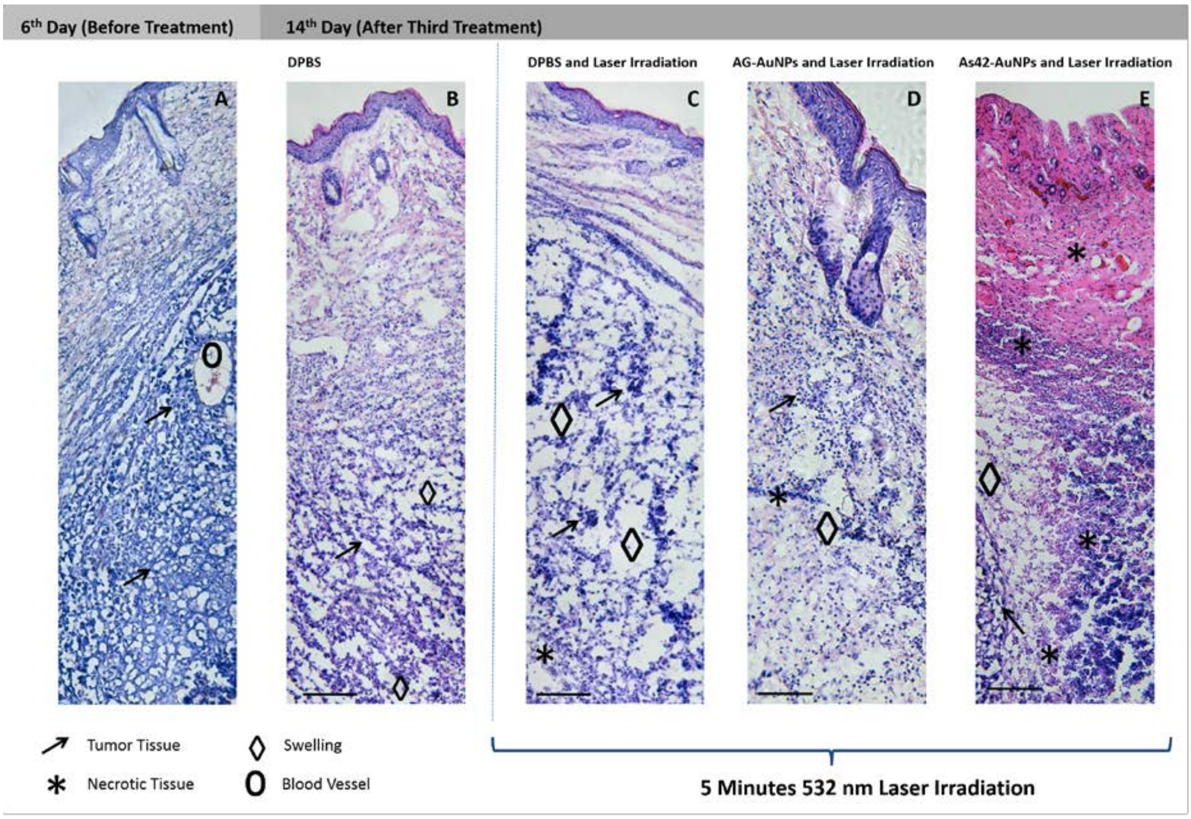

Figure 1S. General view of histopathological changes of solid Ehrlich carcinoma assessed by H&E staining before PPT treatment (A), after tail vein injection of DPBS (B, C), AG-AuNPs (D), AS42-AuNPs (E) after 5 min of laser irradiation at 1.2 Watts. Magnification×50.
