# Supplementary material for: Aptamer-Targeted Plasmonic Photothermal Therapy of Cancer
Source: Mol Ther Nucleic Acids. 2017 Aug 16;9:12–21. doi: 10.1016/j.omtn.2017.08.007 (PMC5582647; doi:10.1016/j.omtn.2017.08.007)
Supplement: Document S2. Article plus Supplemental Information [file mmc2.pdf]

Molecular Therapy
Nucleic Acids
Original Article

Aptamer-Targeted Plasmonic Photothermal Therapy of Cancer

Olga S. Kolovskaya,[1,2] Tatiana N. Zamay,[1,4] Irina V. Belyanina,[1,4] Elena Karlova,[1,3] Irina Garanzha,[1,4] Aleksandr S. Aleksandrovsky,[2,4] Andrey Kirichenko,[1] Anna V. Dubynina,[2] Alexey E. Sokolov,[2,4] Galina S. Zamay,[1,2] Yury E. Glazyrin,[1,2] Sergey Zamay,[2] Tatiana Ivanchenko,[1] Natalia Chanchikova,[1,3] Nikolay Tokarev,[3] Nikolay Shepelevich,[3] Anastasia Ozerskaya,[3] Evgeniy Badrin,[3] Kirill Belugin,[3] Simon Belkin,[3] Vladimir Zabluda,[2] Ana Gargaun,[5] Maxim V. Berezovski,[5] and Anna S. Kichkailo[1,2]

[1]Krasnoyarsk State Medical University named after Professor V.F. Voyno-Yasenetskii, Krasnoyarsk, Russia; [2]Kirensky Institute of Physics, Federal Research Center KSC SB RAS, Krasnoyarsk, Russia; [3]The Federal State-Financed Institution “Federal Siberian Research Clinical Centre under the Federal Medical Biological Agency”, Krasnoyarsk, Russia; [4]Siberian Federal University, Krasnoyarsk, Russia; [5]University of Ottawa, Department of Chemistry and Biomolecular Sciences, Ottawa, ON, Canada

Novel nanoscale bioconjugates combining unique plasmonic photothermal properties of gold nanoparticles (AuNPs) with targeted delivery using cell-specific DNA aptamers have a tremendous potential for medical diagnostics and therapy of many cell-based diseases. In this study, we demonstrate the high anticancer activity of aptamer-conjugated, 37-nm spherical gold nanoparticles toward Ehrlich carcinoma in tumor-bearing mice after photothermal treatment. The synthetic anti-tumor aptamers bring the nanoparticles precisely to the desired cells and selectively eliminate cancer cells after the subsequent laser treatment. To prove tumor eradication, we used positron emission tomography (PET) utilizing radioactive glucose and computer tomography, followed by histological analysis of cancer tissue. Three injections of aptamer-conjugated AuNPs and 5 min of laser irradiations are enough to make the tumor undetectable by PET. Histological analysis proves PET results and shows lower damage of healthy tissue in addition to a higher treatment efficiency and selectivity of the gold nanoparticles functionalized with aptamers in comparison to control experiments using free unconjugated nanoparticles.

INTRODUCTION

Currently, gold nanoparticles (AuNPs) are of a great interest for cancer therapy, especially for thermal destruction of tumor cells, due to their photothermal heating ability under laser irradiation and their ability to be surface functionalized. Anti-cancer thermotherapy is based on the high sensitivity of cancer cells to increased temperature. Heating of a tumor up to +43.5°C leads to irreversible denaturation of proteins, while protein molecules in normal tissue remain intact. AuNPs could serve as “optical heaters” and promote the destruction of cancer cells.[1] Oscillating electric fields of light propagating near a colloidal nanoparticle interact with free electrons, causing a concerted oscillation of an electron charge that is in resonance with the frequency of visible (VIS) or near-infrared (NIR) light. The absorbed light, converted to heat, generates localized hyperthermia and destroys malignant cells. Photothermal therapy (PTT) is currently considered to be a relatively noninvasive and benign alternative for cancer treatment.[2] To deliver nanoparticles to tumor sites, monoclonal antibodies (mAbs) are successfully used for the photodestruction of cancer cells and subsequent cell death.[3] An alternative to antibodies as a nanoparticle delivery vehicle is aptamers. Aptamers are more preferable for the selective delivery of nanoparticles because of their higher stability and lower immunogenicity. Recently, Shi et al. reported a novel activatable theranostic nanoprobe based on aptamers for in vivo cancer imaging and guided PTT.[4] The authors used a DNA aptamer previously selected against the A549 human cancer cell line by Cell-SELEX to functionalize Au@Ag/Au NPs via the thiol-gold bond to achieve specific NIR PTT. Due to the broad absorption spectra of Au@Ag/Au NPs and the expansion of aptamer discovery for varying cancer targets, aptamer-modified gold nanoparticles can be explored for the treatment of other cancer types.

In our study, we demonstrate the PTT activity of aptamer-conjugated 37-nm spherical gold nanoparticles (As42-AuNPs) toward Ehrlich carcinoma cells in tumor-bearing mice (Figure 1). Nanoparticle heating was carried out by irradiation with visible, 536-nm laser light for 5 min. To prove the efficacy of the aptamer-targeted hyperthermia, positron emission tomography (PET) with radioactive glucose and computer tomography (CT) with histopathological analysis of cancer tissue were utilized.

RESULTS AND DISCUSSION

In Vitro Cancer Cell Hyperthermia with As42-AuNPs

AuNPs with an average diameter of 37 nm were used in this study. The absorption spectra, Q(λ), of AuNPs are shown in Figure 2.

Received 13 March 2017; accepted 11 August 2017;
http://dx.doi.org/10.1016/j.omtn.2017.08.007.

Correspondence: Anna S. Kichkailo, Laboratory for Biomolecular and Medical Technologies, Krasnoyarsk State Medical University named after Professor V.F. Voyno-Yasenetskii, 1 P. Zheleznyaka Str., Krasnoyarsk 660022, Russia.
E-mail: aszamay@gmail.com
Correspondence: Maxim V. Berezovski, University of Ottawa, Department of Chemistry and Biomolecular Sciences, Ottawa, ON, Canada.
E-mail: maxim.berezovski@uottawa.ca

 © 2017 The Authors.
This is an open access article under the CC BY license (http://creativecommons.org/licenses/by/4.0/).

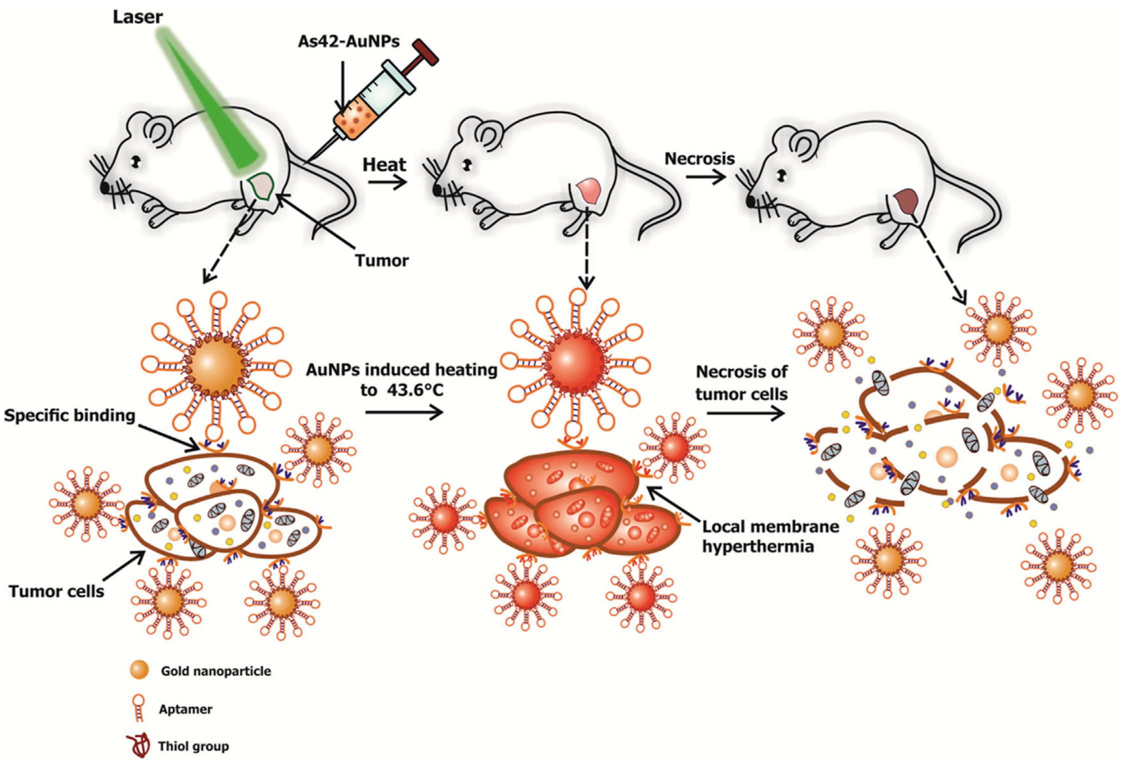

Figure 1. Scheme of Selective Elimination of Cancer Cells In Vivo Using As42-AuNPs in Plasmonic Photothermal Therapy
As42-AuNPs are localized on the tumor cells after injection into a mouse tail vein. Local irradiation of a tumor site with a green laser causes nanoparticle heating and cell death followed by tumor eradication.

Transmission electron microscopy images of AuNPs demonstrate small variations in size and shape (Figure 2, insert). AuNPs were chemically modified with the oligonucleotide containing a disulfide group and subsequently hybridized with either the AS42 aptamer or AG oligonucleotide as a non-specific control. The AS42 aptamer was previously selected by our research group to live Ehrlich's ascites carcinoma cells with a dissociation constant, K_D, of 2.5 nM.[5] A heat shock cognate 71-kDa (HSPA8) protein was identified by protein mass spectrometry as its binding partner. It is important to note that the As42 aptamer has a high affinity for the ascites cells but does not cause apoptosis and does not interfere with cell division.

Therefore, the AS42 aptamer was picked for this study to facilitate targeted delivery of nanoparticles and PTT of cancer cells. Laser irradiation of cancer cells, without nanoparticles, at 532 nm for 10 min did not significantly heat (up to 38.4°C) the cell suspension. However, temperature increased up to 43.6°C for the cells with As42-AuNPs under the same laser irradiation conditions. The photothermal treatment with As42-AuNPs resulted in 45% cell death, measured 3 hr after laser irradiation. This can be attributed to the AS42 aptamer bringing the AuNPs closer to the cancer cell's surface (~100 NPs per cell), after which plasmon resonance caused local membrane hyperthermia, leading to cell death (Figure 3). In control experiments using AG-AuNPs, the heat dissipated in solution because the AG oligonucleotide did not attach AuNPs to the cells. Laser irradiation at 532 nm alone and in the presence of As42 aptamer without nanoparticles did not have an influence on cell viability in vitro. It is interesting to note that nanoparticles modified with AG and As42 decreased the number of dead cells in the absence of irradiation (Figure 3A).

Titration experiments with different concentrations of As42-AuNPs (10, 50, 100, and 200 particles per cell [performed in triplicates]) revealed that 100 As42-AuNPs per cell were enough to kill 83% ± 5% of Ehrlich ascites cells, while 200 particles led to total necrosis in the culture (Figure 3B). Control liver and blood cells did not respond to plasmonic PTT with As42-AuNPs, but the high density of nanoparticles (200 per cell) caused necrosis of 6% ± 3% of the cells (Figure 3C). Therefore, in order to increase the selectivity, a concentration of 100 As42-AuNPs per cell was chosen for further experiments.

The selectivity of the in vitro treatment with the As42-AuNPs has been demonstrated using flow cytometry experiments in a mixture of Ehrlich carcinoma, blood, and liver cells. Before plasmonic photothermal treatment with As42-AuNPs, it was confirmed that Ehrlich carcinoma cells (Figure 4A1), blood and liver cells (Figure 4A2), and their mixture (Figure 4A3) were viable. Two hours after the therapy, the majority of the carcinoma cells became necrotic (Figure 4B1),

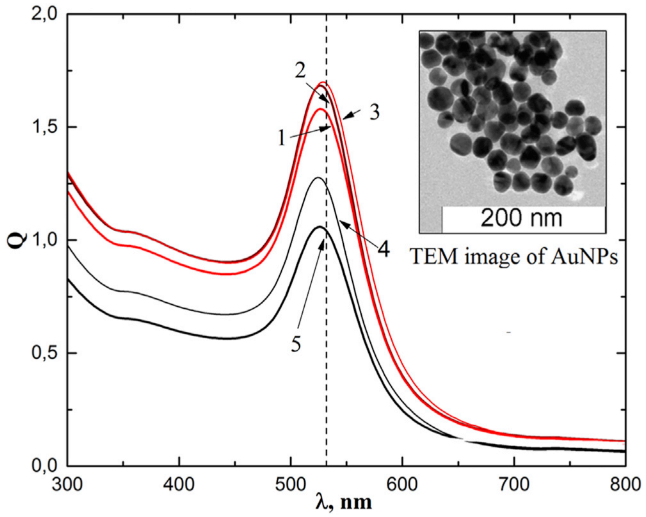

Figure 2. Absorption Spectra of Colloidal Solutions of AuNPs

Curves 1–3 correspond to the following concentrations: $1.1 \cdot 10^{12}$ mL^{-1} AuNPs; 4 – $1.1 \cdot 10^{9}$ mL^{-1} AuNPs; and 5 – $1.1 \cdot 10^{8}$ mL^{-1} AuNPs, respectively. Dashed line indicates the laser wavelength of 532 nm. Insert: transmission electron microscopy (TEM) image of AuNPs.

while most of the liver and blood cells stayed live (Figure 4B2). In the mixture, carcinoma cells died, while liver and blood cells remained viable (Figure 4B3).

Plasmonic PTT with As42-AuNPs in Mice

Mice implanted with solid Ehrlich carcinoma underwent PTT every second day, starting from day 7 after tumor transplantation until day 11. Changes in temperature at the surface of tumors were controlled using thermography (Figure 5A). Interestingly, 532-nm laser irradiation increased the temperature of the tumors in mice treated with oligonucleotide-modified gold nanoparticles up to 46°C, while mice injected with Dulbecco's PBS (DPBS) experienced a temperature rise of more than 40°C. This is evidence for specific and non-specific AuNPs concentrating at the tumor. Despite the similar temperature increase in the case of As42-AuNP and AG-AuNP treatments, aptamer-modified Au-NPs appeared to be more effective because of their targeted delivery of AuNPs to the surface of tumor cells. Changes in hip girth and tumor size, caused by the thermal photoablation, depended on the injected substance and are shown in Figure 5B. Hips of the mice injected with DPBS grew exponentially, as expected. Additional laser irradiation resulted in increased tumor development during the course of the experiment (Figure 5B). This might be due to heating of the tumor, up to 40°C, by the laser. The administration of AG-AuNPs followed by irradiation at 532 nm decreased tumor size in the subsequent group. Using As42-AuNPs increased the effects of laser irradiation (Figure 4B3). Administering As42-AuNPs without laser exposure slows down tumor growth, somehow hindering cancer cell division. Necrotic changes in treated and non-treated tumors were observed on day 13 after tumor transplantation (Figure 5C).

In order to control the last stages of tumor development after treatment, we performed PET combined with CT (PET/CT) for one mouse from each experimental group, since, visually, tumors treated the same way were similar, and the girth of hips in relation to the tumors did not differ. Histopathological examination of the tumors was performed for every experimental mouse.

Since Ehrlich carcinoma cells actively consume glucose, the viability and functional state of this tumor could be assessed with the help of the radionuclide method of functional diagnostics (PET/CT) using the radiopharmaceutical [^{18}F]-fluorodeoxyglucose. [^{18}F]-fluorodeoxyglucose is a biological analog of glucose and penetrates from the vascular into the extracellular space and then into the cells, where it is phosphorylated by hexokinase. The reaction product is [^{18}F]-deoxyglucose-6-phosphate. Unlike glucose phosphate, [^{18}F]-deoxyglucose-6-phosphate does not enter into further reactions and remained in the cells during the study, which allowed the accumulation and measurement of the radionuclide in the growing tumor.[6]

On day 6 after tumor transplantation and before the AuNP treatment and laser irradiation, the tumor showed a 17 mm × 15 mm homogeneous structure with tissue density (45–50 Hounsfield units [HU]) and actively accumulated [^{18}F]- fluorodeoxyglucose (Figure 6A). The glucose was also accumulated in the bladder and liver of this mouse. Histopathological examination of untreated tumors showed that the tumors of all mice had homogeneous solid structures throughout the volume; inflammatory response was low, carcinoma cells infiltrated and replaced the skeletal muscle tissue, and the tumor recruited new blood vessels (Figure S1A).

In a control mouse after injection of DPBS, the tumor grew 25% more, reaching a size of 23 mm × 19 mm on day 14. Its structure was non-uniform, and density varied from 25 to 50 HU, with indistinct contours and a high level of accumulation of radiopharmaceutical [^{18}F]-fluorodeoxyglucose (Figure 6B). Moreover, a metastasis in the testicle of the examined mouse was detected. The bladder of this mouse was empty, so there was no glucose accumulation (Figure 6B). Histopathological analyses of tumors in the hips revealed relatively intact tumors (Figure S1B) composed of viable atypical cells with pleomorphic nuclei of different shapes and cytoplasmic volumes (Figures 7A and 7B).

After the third treatment with DPBS injection, followed by laser irradiation in the right leg, the tumor had the size of 20 mm × 17 mm, and its large part (16 mm × 10 mm) presented a non-metabolic necrotic area (Figure 6C). Radiopharmaceutical accumulation was 4–5 mm in diameter, located in the upper inner part of the tumor. [^{18}F]-fluorodeoxyglucose was also accumulated in the bladder of this mouse (Figure 6C). Tumors of all experimental mice were massive with swelling, small ulcers (Figure 5C). Tissues had heterogeneous structures (Figure S1C), sites of necrosis with complete loss of tissue structure, but without sufficient inflammation (Figures 7C and 7D). Probably, the lack of new blood vessels in the tumor and laser heating to 40°C resulted in necrotic changes.

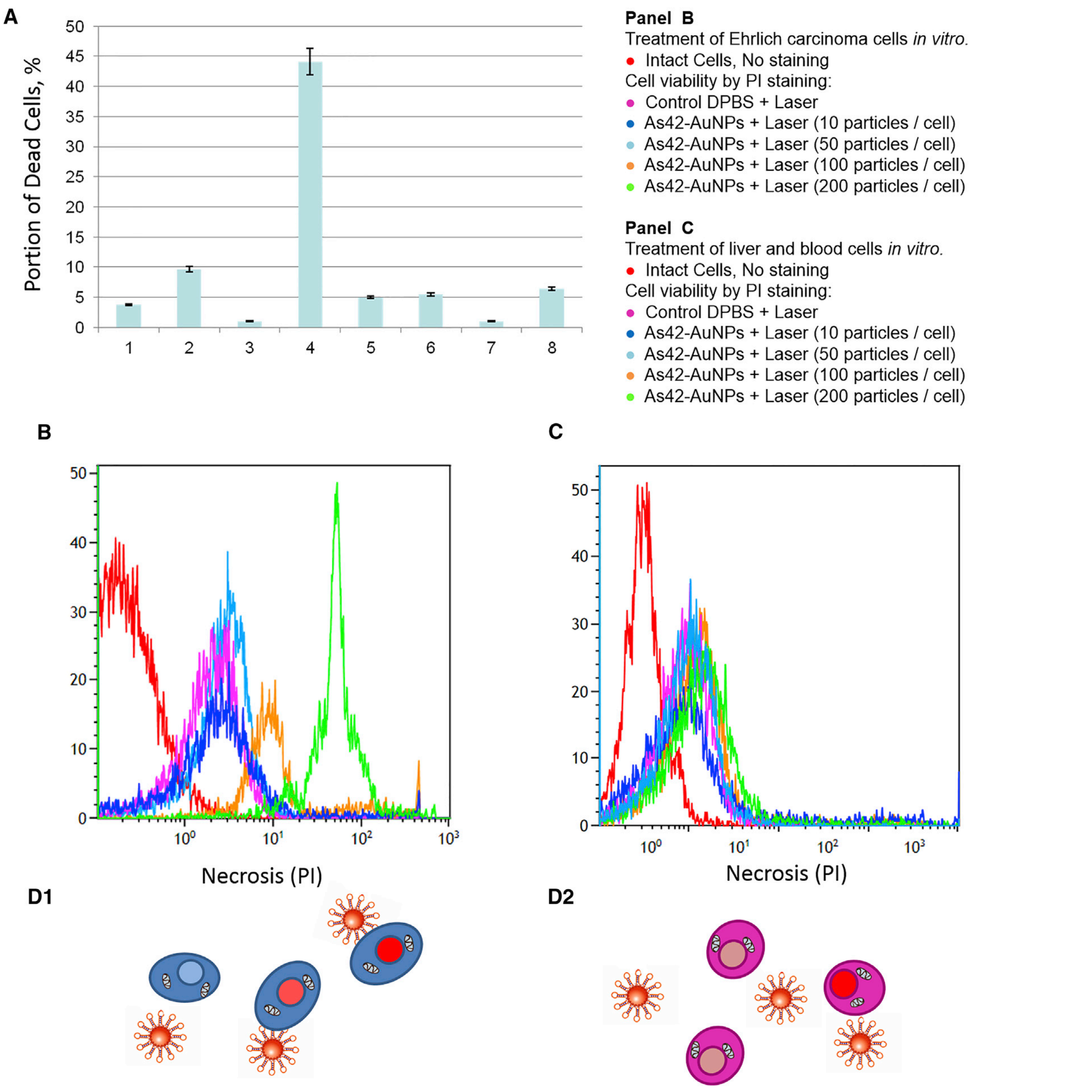

Figure 3. Effects of Photothermal Therapy of Ehrlich Carcinoma Cells Depending on the Presence of Gold Nanoparticles and/or DNA Aptamers In Vitro

(A) Portions of dead cells were measured using trypan blue 3 hr after treatment in different experimental models. 1, intact Ehrlich carcinoma cells; 2, Ehrlich carcinoma cells after a 4-min laser irradiation; 3, Ehrlich carcinoma cells incubated with As42-AuNPs; 4, Ehrlich carcinoma cells preincubated with As42-AuNPs after 4 min of irradiation; 5, Ehrlich carcinoma cells incubated with free aptamer As42; 6, Ehrlich carcinoma cells incubated with free aptamer As42 after 10 min of irradiation; 7, Ehrlich carcinoma cells incubated with AG-AuNPs; 8, Ehrlich carcinoma cells incubated with AG-AuNPs after 4 min of irradiation. (B) Viability of Ehrlich cells after plasmonic photothermal therapy in vitro with As42-AuNPs (in the ratios of 10, 50, 100, and 200 AuNPs per cell). (C) Viability of liver and blood cell mixture after plasmonic photothermal therapy in vitro with As42-AuNPs (in the ratios of 10, 50, 100, and 200 AuNPs per cell). PI, propidium iodide. (D) Schematic representation of the Ehrlich, liver, and blood cell viability measurements after plasmonic photothermal treatment. All data are presented as the mean ± SEM.

Three treatment procedures with non-specific oligonucleotide AG-AuNPs followed by 532-nm laser irradiation resulted in visual tumor size reduction. PET indicated that the tumor was reduced in size by 18%, to 14 mm × 12 mm, with a density of 35–42 HU (Figure 6D). The tumor had a heterogeneous structure and moderate swelling (Figure S1D). All mice in this group had ulcerative defects (Figures 5CIII and 7E) and necrotic changes located under skin dermis (Figure 7E). In the center of the tumor, necrotic areas were found in the form of islands with completely destructed cells (Figure 7F). Dead (Figure 7G) and viable (Figure 7H) leukocytes

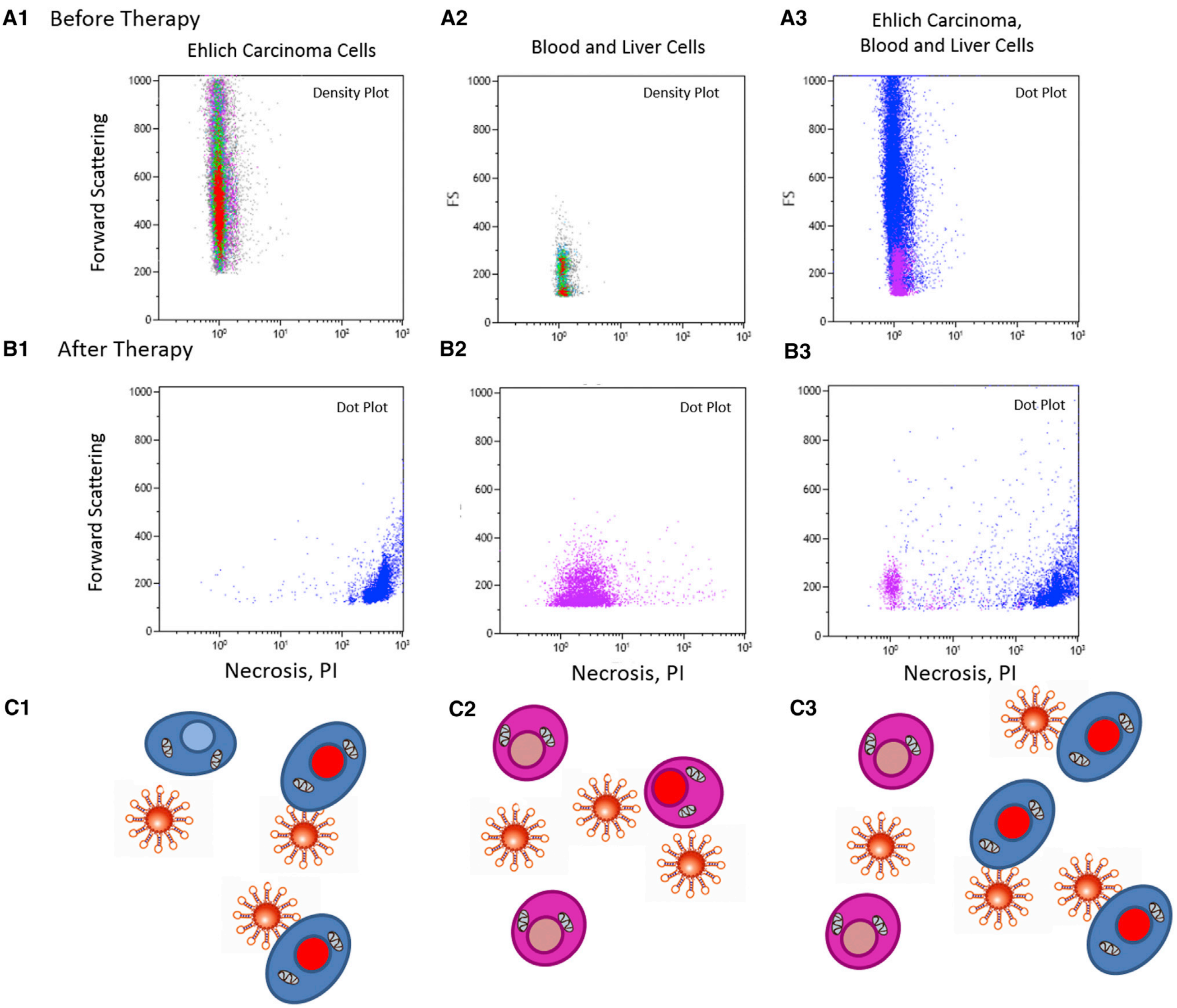

Figure 4. Selectivity of Plasmonic Photothermal Therapy In Vitro

(A) Necrosis in (1) intact Ehrlich carcinoma cells; (2) a liver and blood cell mixture (flow cytometry density plots); and (3) a mixture of Ehrlich carcinoma, liver and blood cells (flow cytometry dot plot). (B) Necrosis in (1) Ehrlich carcinoma cells; (2) a liver and blood cell mixture; and (3) a mixtue of Ehrlich carcinoma, liver, and blood cells (flow cytometry dot plots) after plasmonic photothermal therapy. (C) Schematic representation of (C1) necrotic Ehrlich carcinoma cells; (C2) intact liver and blood cells; and (C3) a mixture of Ehrlich carcinoma, liver, and blood cells after photothermal treatment.

dominated in the inflammatory infiltrates and surrounded the viable carcinoma areas.

The treatments with As42-AuNPs and laser irradiation eliminated tumors almost completely, as seen in PET/CT images (Figure 6E). All mice had big ulcers in the tumor sites. Based on histopathological analysis, the tumor sites were mostly necrotic, with high leukocyte infiltration (Figure 7K). Figure 7 shows therapeutic effects in details: necrosis of the skin and underlying tumor, the loss of the epidermis, and bleeding of the dermis (Figures 7I–7K). Dead cancer cells had the characteristic signs of karyopyknosis, karyorhexis, and autolysis (Figure 7L). Although in the center, the tumor looked viable in the histological sections (Figure S1E), it was not metabolically active; according to PET analyses, the cells did not accumulate [^{18}F]-fluorodeoxyglucose (Figure 6E).

Some nonspecific accumulation of [^{18}F]-fluorodeoxyglucose was observed near the mouse tail vein and bladder. This is due to the administration of the radiopharmaceutical into the mouse tail vein in a physiological saline solution that is excreted in urine.

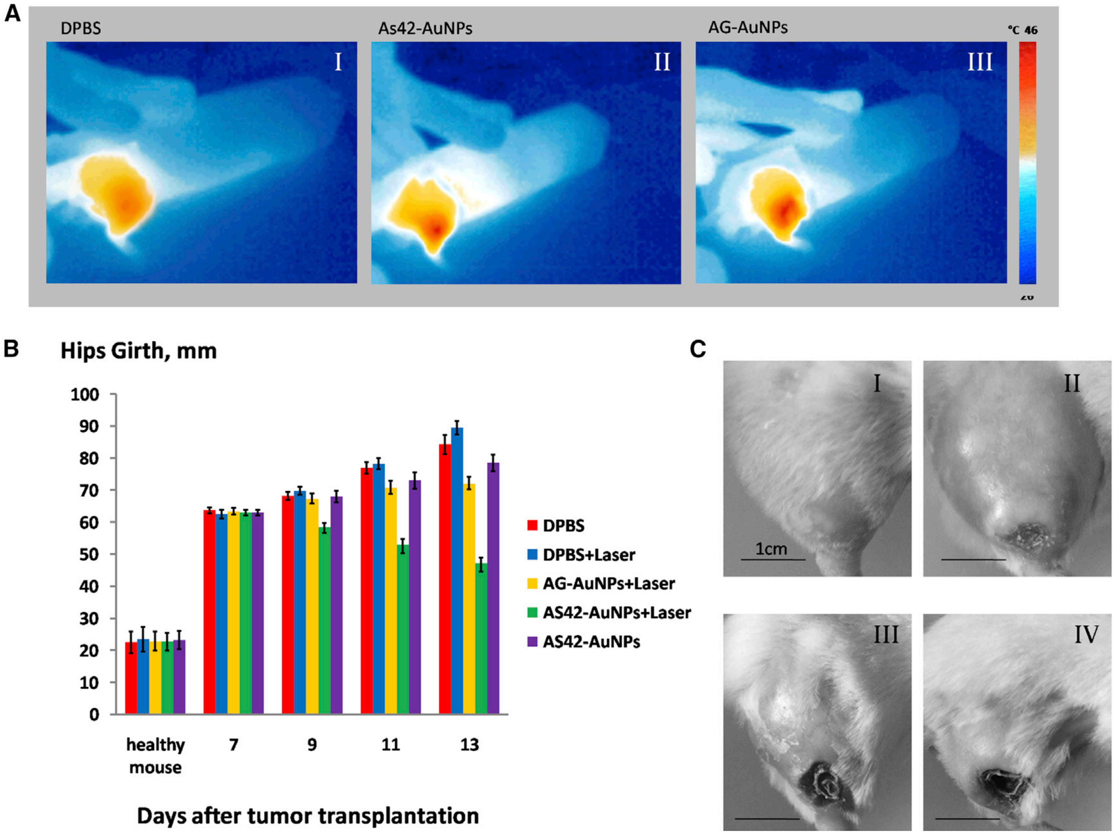

Figure 5. Targeted Plasmonic Photothermal Therapy In Vivo

(A) Thermal images of mouse hips after tail-vein injection of DPBS (AI), AG-AuNPs (AII) modified, or AS42-AuNPs (AIII) after 5 min of laser irradiation at 1.2 W. (B) Changes in the hip girth within tumors. The treatment has been performed on days 7, 9, and 11. (C) The representative images of the tumors of treated and non-treated mice are on day 11 treated with (I) DPBS only; (II) DPBS and 5 min of laser irradiation, (III) AG-AuNPs and 5 min of laser irradiation (IV) AS42-AuNPs and 5 min of laser irradiation. All data are presented as the mean ± SEM.

Hepatotoxicity of As42-AuNPs was monitored by measuring standard blood serum biochemistry parameters such as cholesterol, serum alanine amino-transferase (ALT), alkaline phosphatase (AST), and bilirubin (Table 1).[7] ALT is involved in energy metabolism in liver, total bilirubin is a marker of hepatobiliary injury and hemolysis, and ALP indicates hepatocyte damage. Healthy male and female mice underwent three treatment procedures every other day with As42-AuNPs. This did not cause significant changes in blood biochemical parameters, compared to the control group treated with DPBS, and did not depend on gender. Inflammation and hydration status after the treatment with nanoparticles were evaluated by the total protein concentration (Table 1). All tested parameters indicated that treatment with As42-AuNPs was safe and did not cause unwanted hepatotoxic effects.

DISCUSSION

Plasmonic PTT represents the least invasive method of malignant neoplasia treatment. This method uses the conversion of the photon's energy into thermal energy sufficient for tumor destruction. The use of hyperthermia is the most promising for cases when surgical removal of a tumor is challenging. Our study shows photothermal destruction of Ehrlich's ascites carcinoma cells using AuNPs functionalized with DNA aptamers. Using the tumor-specific aptamers enables precise delivery of AuNPs to the membrane of a target cells and enhances the specificity of photothermotherapy. Laser irradiation resonantly excites plasmons and produces the heating effect of AuNPs, causing damage to cancer target cells while leaving healthy cells untouched. One can expect aptamer-functionalized AuNPs to bind selectively to cancer cells and result in the localization of AuNPs within a tumor, which is necessary for the enhancement of therapeutic treatment efficiency and suppression of lateral negative effects, such as possible overheating of the healthy cells and tissues.

Aptamer-functionalized AuNPs possess a series of additional advantages; particularly, they are stable against aggregation by negative charges of oligonucleotides, and they do not form conglomerates inside blood vessels. Moreover, in contrast to protein antibodies, aptamer-functionalized AuNPs are practically non-immunogenic, and any loss of confirmation that such drugs might experience due to storage conditions can be easily reversed with no loss in quality.

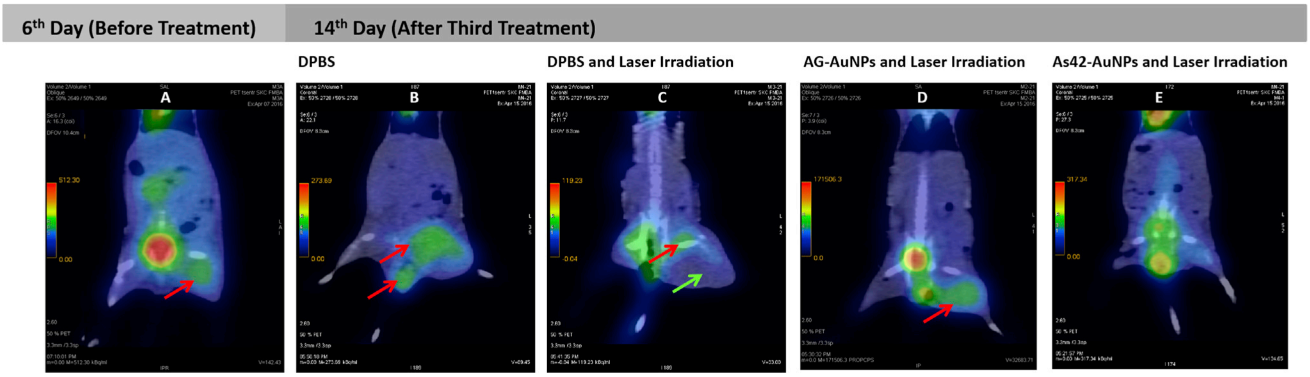

Figure 6. PET/CT and Histopathological Images of Mice after PPT Treatments

(A–E) PET/CT images of mice before PPT treatment (A); after treatment with DPBS without laser irradiation (B); after PPT therapy with DPBS (C), AG-AuNPs (D), and AS42-AuNPs (E). Red arrows indicate accumulation of ^{18}[F]-fluorodeoxyglucose; green arrow indicate necrosis and swelling.

Despite the aforementioned advantages, we faced some drawbacks related to the fact that tumor cells, as we proved, are destructed via necrosis, which led to high levels of inflammation in the organism. Adjusting the power and selection of a softer regime of laser irradiation allows finding a gentler mode of laser thermal destruction. In this mode, tumor cells die slowly without the development of the hyperactive inflammatory process.

In conclusion, the present work demonstrates, for the first time, the use of AuNPs functionalized by aptamers for plasmonic PTT. Further research can focus on the development of the particles possessing NIR plasmon resonance for deeper treatment of tumors.

MATERIALS AND METHODS

Chemicals and Materials

DNA probes were custom designed and synthesized by Integrated DNA Technologies, USA. DPBS, CELLSTAR in DMEM, trypan blue, and fetal bovine serum (FBS) were purchased from Sigma-Aldrich, USA. AuNPs, with an average diameter of 37 nm, were purchased from BioTest, Novosibirsk, Russia.

Animals

Male white, 6-week-old 25 g Imprinting Control Region (ICR) mice were purchased from Siberian Federal University, Krasnoyarsk, Russia. Tumor-bearing animals were prepared through intramuscular injection of two million Ehrlich carcinoma cells into the right leg of the mice. Tumors were then allowed to grow for 5 days. On days 5, 7, and 9 after the tumor transplantation using 2×10^6 Ehrlich carcinoma cells, all animals were treated with aptamer-functionalized, gold-coated magnetic nanoparticles.

This study was carried out in strict accordance with the recommendations in the NIH Guide for the Care and Use of Laboratory Animals. The protocol was approved by the Local Committee on the Ethics of Animal Experiments of the Krasnoyarsk State Medical University. All procedures were performed under anesthesia, and all efforts were made to minimize the suffering of the animals.

Cell Culture

For the in vitro studies, a mouse Ehrlich carcinoma cell culture was utilized. The mouse ascites cells were cultured in 35× 10-mm cell-culture dishes (CELLSTAR) in DMEM supplemented with 100 U mL^{-1} penicillin, 100 U mL^{-1} streptomycin, and 5% (v/v) FBS in a humidified atmosphere containing 5% CO_2 at 37°C. All cell experiments were performed in DPBS containing 0.9 mM $CaCl_2$ and 0.49 mM $MgCl_2$.

Functionalization of Gold Nanoparticles with DNA Aptamers

AuNPs with an average diameter of 37 nm were used in this study. Extinction spectra of conjugates of AuNPs with the DNA aptamer were recorded using a UV-3600 spectrophotometer (Shimadzu, Japan).

Nanoparticle stabilization was carried out with a thiolated probe: a high-performance liquid chromatography (HPLC)-purified oligonucleotide 5′-CGT GGT TAC AGT CAG AGG AGA A-/ThioMC6-D/-3′ modified at the 3′ end with a 6-hydroxyhexyl disulfide group (Integrated DNA Technologies, USA) in AuNP storage buffer for 24 hr at 4°C on a shaker (final concentration, 500 nM). This thiolated probe was complimented to the 5′ end of the AS42 aptamer: 5′-CTC CTC TGA CTG TAA CCA CGT CAA TGG GTG ATA TAT GCA GGT TAC GCT GGC TAG TTG AAA GCA TAG GTA GTC CAG AAG CC-3′. This mixture was diluted 2 times by mixing it with DPBS (with calcium and magnesium) and then with an equimolar amount of the AS42 aptamer or the AG oligonucleotide: 5′-CTC CTC TGA CTG TAA CCA CG $(AG)_{20}$ GCA TAG GTA GTC CAG AAG CC-3′ as a nonspecific control and incubated for an additional 24 hr on a shaker. Prior to use, oligonucleotides were heated at 95°C for 10 min and cooled on ice for 10 min.

In Vitro Analyses of PTT

One million mouse Ehrlich carcinoma cells in 1 mL colorless high-glucose DMEM were used for the in vitro analyses.

A continuous-wave Millennia V laser (Spectra-Physics Lasers, USA) was used for excitation of plasmon resonance of AuNPs at 532 nm.

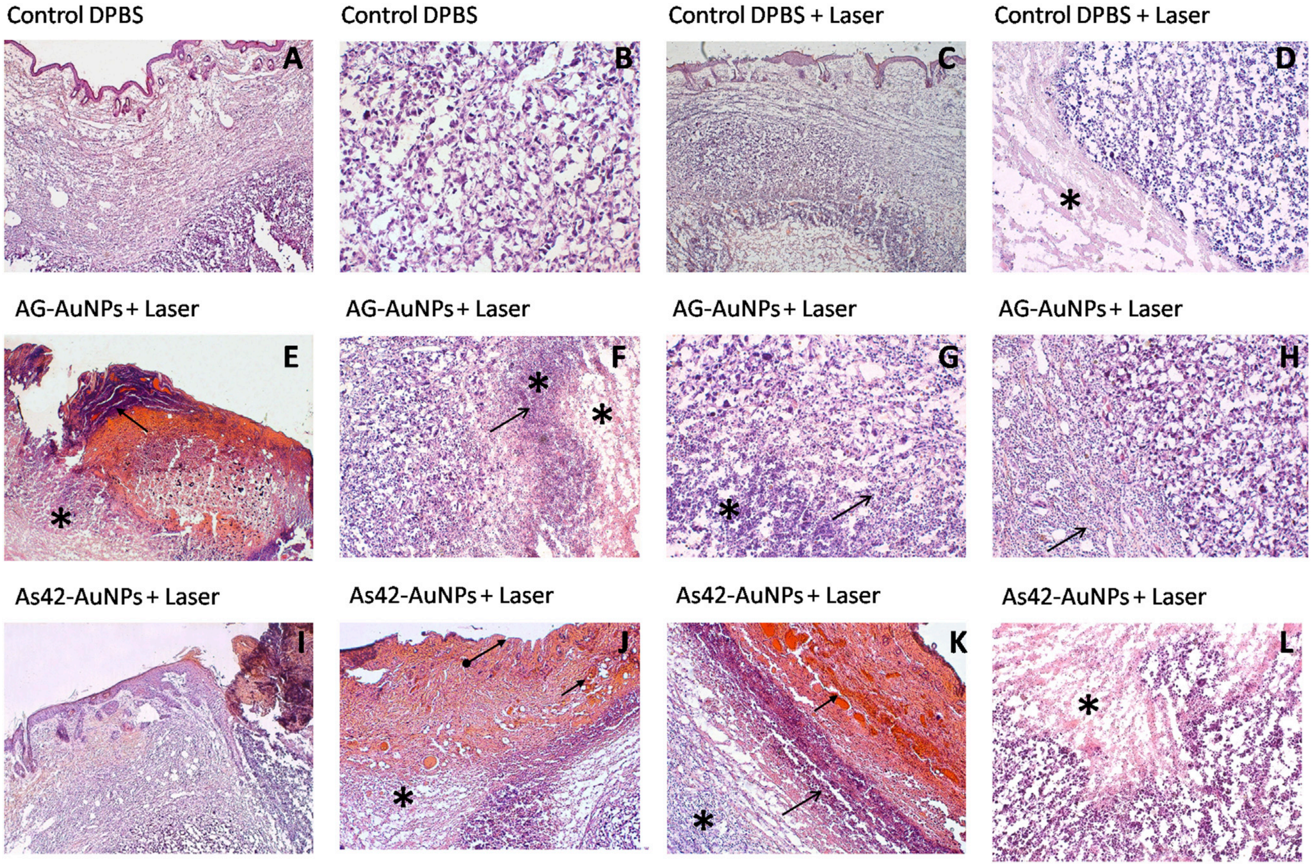

Figure 7. Histopathological Assessment of Solid Ehrlich Carcinoma Tumor

H&E staining. Representative sections were obtained after PPT with DPBS only. (A) Carcinoma under epidermis. (B) General view of mouse carcinoma treated with DPBS only. Magnification, 200×. PPT with DPBS and 5 min of laser irradiation. (C) General view of the tumor under epidermis. (D) The border between relatively intact viable and necrotic (asterisk) tumor tissues lacking inflammatory cells. Magnification, 100×. PPT with AG-AuNPs and 5 min of laser irradiation. (E) Ulcerative defect. (F) Scab (arrow) in the bottom of the wound and tumor necrosis under the dermis (asterisk). Magnification, 50×. (G) Complete destruction of tumor tissue in the center of the necrotic area (asterisk). Mostly dead segmented leukocytes (arrow). Magnification, ×100. (H) Inflammatory infiltration of segmented leukocytes at the tumor border (arrow). Magnification, 100×. PPT with As42-AuNPs and 5 min of laser irradiation. (I) Ulcerative defect, carcinoma necrosis (asterisk) in the bottom under the wound. (J) Necrosis of the skin and underlying tumor (asterisk), the loss of the epidermis (arrow), and the dermis bleeding (arrows). Magnification, 50×. (K) The boundary of the tumor necrosis in the dermis is separated with the area of leukocyte infiltration (arrow), outside of which hemocirculatory disorder takes place. Magnification, 50×. (L) Tumor necrosis (asterisk) is characterized by karyopyknosis, karyorhexis, and autolysis. Magnification, 100×.

The cuvette with the solution was placed in the region where the diameter of the laser beam was equal to 2 cm; the temperature of the suspension was monitored using a thermal imager. Laser power was set to be 2.5 W, and exposition time was 10 min per session. Eight samples containing 10^6 cells were prepared and treated as follows: intact cells (10^6 cells per milliliter) in 1 mL; cells after 10-min laser irradiation at a 532-nm wavelength; cells incubated with As42-AuNPs (10^8 particles per milliliter); cells incubated with As42-AuNPs (108 particles per milliliter) for 30 min followed by 10-min laser irradiation at a 532-nm wavelength; cells incubated with the aptamer As42 (500 nM) for 30 min; cells incubated with the aptamer As42 (500 nM) for 30 min followed by 10-min laser irradiation at a 532-nm wavelength; cells incubated with AG-AuNPs (10^8 particles per milliliter); and cells incubated with AG-AuNPs (10^8 particles per milliliter), followed by 10-min laser irradiation at a 532-nm wavelength. The samples with the cells were incubated at 37°C in a humidified atmosphere containing 5% CO_2 before and after irradiation. All samples were prepared in three replicates.

Determination of the percentage of damaged cells was performed 3 hr after the laser irradiation using 0.4% trypan blue (Sigma-Aldrich, USA).

In Vivo Analyses of PTT

Six-week-old, 25-g ICR male mice were used in this study; five animals per group. For 2 weeks before the experiments, animals were trained to stay calm when being handled and inside the test cage. Two million Ehrlich carcinoma cells were transplanted into the right

Table 1. Blood Serum Biochemistry Parameters Performed for Male and Female Mice Treated with As42-AuNPs in DPBS or with DPBS Alone

Sample	Cholesterol (mmol/L^{-1})	Total Protein (g/L^{-1})	Alanine Amino-Transferase (IU/L^{-1})	Alkaline Phosphatase (IU/L^{-1})	Total Bilirubin (μmol/L^{-1})
As42-AuNPs					
Female (N = 5)	1.49 ± 0.33	47.85 ± 3.46	18.40 ± 5.67	263.30 ± 94.81	5.90 ± 0.51
Male (N = 5)	1.91 ± 0.50	49.83 ± 5.21	21.1 ± 7.17	289.76 ± 103.09	6.01 ± 0.41
DPBS					
Female (N = 5)	2.0 ± 0.30	52.09 ± 1.28	14.05 ± 5.73	206.61 ± 57.13	6.06 ± 0.58
Male (N = 5)	2.20 ± 0.28	56.10 ± 2.06	20.03 ± 6.01	256.30 ± 78.25	6.30 ± 0.65

All data are presented as the mean ± SEM.

leg of each mouse. Every second day, starting from day 7 after tumor transplantation until day 11, animals underwent PTT. A continuous-wave Millennia V laser was used for excitation of plasmon resonance of AuNPs at 532 nm. Fur from the mice legs with the tumors were removed using Veet hair removal cream (Reckitt Benkiser, France). The animal was placed into an experimental cage with the tumor leg outside the region where the diameter of the laser beam was equal to 2 cm; the temperature of the leg was monitored using an infrared camera (Testo 875-1i from TESTO, Germany). Laser power was set to be 2.5 W, and exposition time was 5 min per session.

Tail-vein injections for the mice randomly divided into five groups (on days 7, 9, and 11 after the tumor transplantation, 3 times total) were as follows:

Group 1: injection of 100 μL DPBS;

Group 2: injection of 100 μL DPBS followed by 5-min laser irradiation;

Group 3: injection of As42-AuNPs in 100 μL DPBS (1.1 μg/kg^{-1}; ~50 particles per cell);

Group 4: injection of As42-AuNPs in 100 μL DPBS (1.1 μg/kg^{-1}; ~50 particles per cell) followed by 5-min laser irradiation; and

Group 5: injection of AG-AuNPs in 100 μL DPBS (1.1 μg/kg^{-1}; ~50 particles per cell) followed by 5-min laser irradiation.

After 30 min, animals were placed inside the test cage, and the leg with the tumor stayed outside and was irradiated with the 532-nm laser for 5 min.

PET/CT

Tumor volumes were monitored using PET/CT. On day 6 (before the first treatment) and day 12 after tumor transplantation (the next day after the third treatment procedure), animals were injected intravenously with 4–6 MBq (0.11–0.16 mCi) [^{18}F]-fluorodeoxyglucose, followed by a PET/CT scan 1 hr after injection. Animals under anesthesia (ketamine/xylazine: intraperitoneal dose at 70 mg/kg of ketamine and 5–12 mg/kg xylazine) were fixed for imaging. The study was conducted with a Discovery PET/CT 600 scanner (General Electric, USA) and consisted of CT in a spiral mode with 3.75-mm layer thickness followed by post-reconstruction with a 0.625-mm slice. Afterward, positron emission scanning was done in 3D mode for 5 min with iterative reconstruction of the acquired images. Obtained data were analyzed using PET VV software at an AW Volume Share 5 work station. Efficiency of the treatment was estimated by tumor localizations, contours, sizes, and the degree of [^{18}F]-fluorodeoxyglucose accumulation. CT images were analyzed using the Hounsfield densitometry scale.

Tissue Analysis

Microscopy analyses of the tumor tissue sections were performed in order to evaluate histological changes of the tumors. Tumors were harvested and placed in 3.7% formalin on the day following the last treatment procedure. Tumor tissue sections for staining were prepared using the HM 525 cryostat. The tissue sections were fixed onto a glass polylysine slide and stained with H&E dyes by the standard Blick method. Finally, sections were imaged with an Axio Imager A1 optical microscope and an AxioCam MRc 5 high-resolution camera (Carl Zeiss, Germany). Magnification was 50×, 100×, 200×, and 400×.

In Vivo Toxicity of Aptamer-Modified AuNPs

Six-week-old, 25-g ICR male mice were used in this study; 10 animals per group. The tail-vein injections on the mice (5 female and 5 male in each group) were performed on days 1, 3, and 5 (3 times total) were as follows:

Group 1: injections of As42-GMNPs in 100 μL DPBS (1.6 μg/kg); and

Group 2: injections of 100 μL DPBS.

Toxicity was estimated by the changes in blood biochemistry (cholesterol, total protein, ALT, AST, and bilirubin); this was performed using the COBAS INTEGRA 400 plus analyzer (Roche Diagnostics, Switzerland). Parameters for male and female mice were analyzed separately. All data were presented as the mean ± SEM.

Identification of Aptamer's Binding Partner

Identification of the binding partner of the aptamer As42 was done using a protocol previously demonstrated by Zamay et al.[8] In brief,

protein binding partners have been purified from the cell lysate using magnetic separation with 1 mg Streptavidin MagneSphere Paramagnetic Particles (Promega, USA) and identified by mass spectrometric analysis of 10 mL protein-digest using nanoflow ultra-high-pressure liquid chromatography (Easy-nLC 1000, Thermo Scientific) and tandem mass spectrometry with an Orbitrap Velos Pro mass spectrometer (Thermo Scientific). Mathematical analyses were done with the Proteome Discoverer 1.3 software, Sequest search engine and SwissProt database, and MaxQuant 1.4 proteomic software. Experiments were performed in triplicates.

SUPPLEMENTAL INFORMATION

Supplemental Information includes one figure and can be found with this article online at http://dx.doi.org/10.1016/j.omtn.2017.08.007.

AUTHOR CONTRIBUTIONS

T.N.Z. and A.S.K. conceived and designed the experiments. O.S.K., T.N.Z., I.V.B., and G.S.Z. performed animal and cell experiments. D.V.A. and I.G. performed toxicity experiments. A.K. and T.I. performed histology experiments. Y.E.G. performed mass spectrometry identification. E.K., N.C., N.T., N.S., A.O., E.B., K.B., and S.B. performed PET. A.S.A., V.Z., and A.E.S. performed laser irradiation and nanoparticle characterization. O.S.K., T.N.Z., A.G., M.V.B, and A.S.K. wrote and edited the manuscript. S.Z. contributed the general idea and supervising. All authors reviewed the manuscript.

CONFLICTS OF INTEREST

The authors declare no competing financial interests.

ACKNOWLEDGMENTS

We thank Mr. Yousef Risha for improving the use of English in the manuscript and Mr. George Y. Vorogeikin, Mr. Yuri I. Vorogeikin, and “OKB ART” for the infrared imaging. This research is supported by Ministry of Education and Science Federal Target Program #14.607.21.0104 (RFMEFI60714X0104).

REFERENCES

1. Lapotko, D.O., Lukianova, E., and Oraevsky, A.A. (2006). Selective laser nano-thermolysis of human leukemia cells with microbubbles generated around clusters of gold nanoparticles. Lasers Surg. Med. *38*, 631–642.

2. Lavik, E., and von Recum, H. (2011). The role of nanomaterials in translational medicine. ACS Nano *5*, 3419–3424.

3. Zharov, V.P., Galitovskaya, E.N., Johnson, C., and Kelly, T. (2005). Synergistic enhancement of selective nanophotothermolysis with gold nanoclusters: potential for cancer therapy. Lasers Surg. Med. *37*, 219–226.

4. Shi, H., Ye, X., He, X., Wang, K., Cui, W., He, D., II, Li, D., and Jia, X. (2014). Au@Ag/Au nanoparticles assembled with activatable aptamer probes as smart “nano-doctors” for image-guided cancer thermotherapy. Nanoscale *6*, 8754–8761.

5. Kolovskaya, O.S., Zamay, T.N., Zamay, A.S., Glazyrin, Y.E., Spivak, E.A., Zubkova, O.A., Kadkina, A.V., Erkaev, E.N., Zamay, G.S., Savitskaya, A.G., et al. (2014). DNA-aptamer/protein interaction as a cause of apoptosis and arrest of proliferation in Ehrlich ascites carcinoma cells. Biochemistry (Mosc). Suppl. Ser. A Membr. Cell Biol. *8* (1), 60–72.

6. Gallagher, B.M., Fowler, J.S., Gutterson, N.I., MacGregor, R.R., Wan, C.N., and Wolf, A.P. (1978). Metabolic trapping as a principle of oradiopharmaceutical design: some factors resposible for the biodistribution of [18F] 2-deoxy-2-fluoro-D-glucose. J. Nucl. Med. *19*, 1154–1161.

7. Hayes, A.W., and Kruger, C.L. (2014). Detection and evaluation of chemically induced liver injury. In Hayes’ Principles and Methods of Toxicology, 6th edition, G.L. Plaa, M. Charbonneau, and I. Plante, eds. (CRC Press), pp. 2156–2157.

8. Zamay, G.S., Ivanchenko, T.I., Zamay, T.N., Grigorieva, V.L., Glazyrin, Y.E., Kolovskaya, O.S., Garanzha, I.V., Barinov, A.A., Krat, A.V., Mironov, G.G., et al. (2017). DNA aptamers for the characterization of histological structure of lung adenocarcinoma. Mol. Ther. Nucleic Acids *6*, 150–162.

OMTN, Volume 9

Supplemental Information

Aptamer-Targeted Plasmonic Photothermal Therapy of Cancer

Olga S. Kolovskaya, Tatiana N. Zamay, Irina V. Belyanina, Elena Karlova, Irina Garanzha, Aleksandr S. Aleksandrovsky, Andrey Kirichenko, Anna V. Dubynina, Alexey E. Sokolov, Galina S. Zamay, Yury E. Glazyrin, Sergey Zamay, Tatiana Ivanchenko, Natalia Chanchikova, Nikolay Tokarev, Nikolay Shepelevich, Anastasia Ozerskaya, Evgeniy Badrin, Kirill Belugin, Simon Belkin, Vladimir Zabluda, Ana Gargaun, Maxim V. Berezovski, and Anna S. Kichkailo

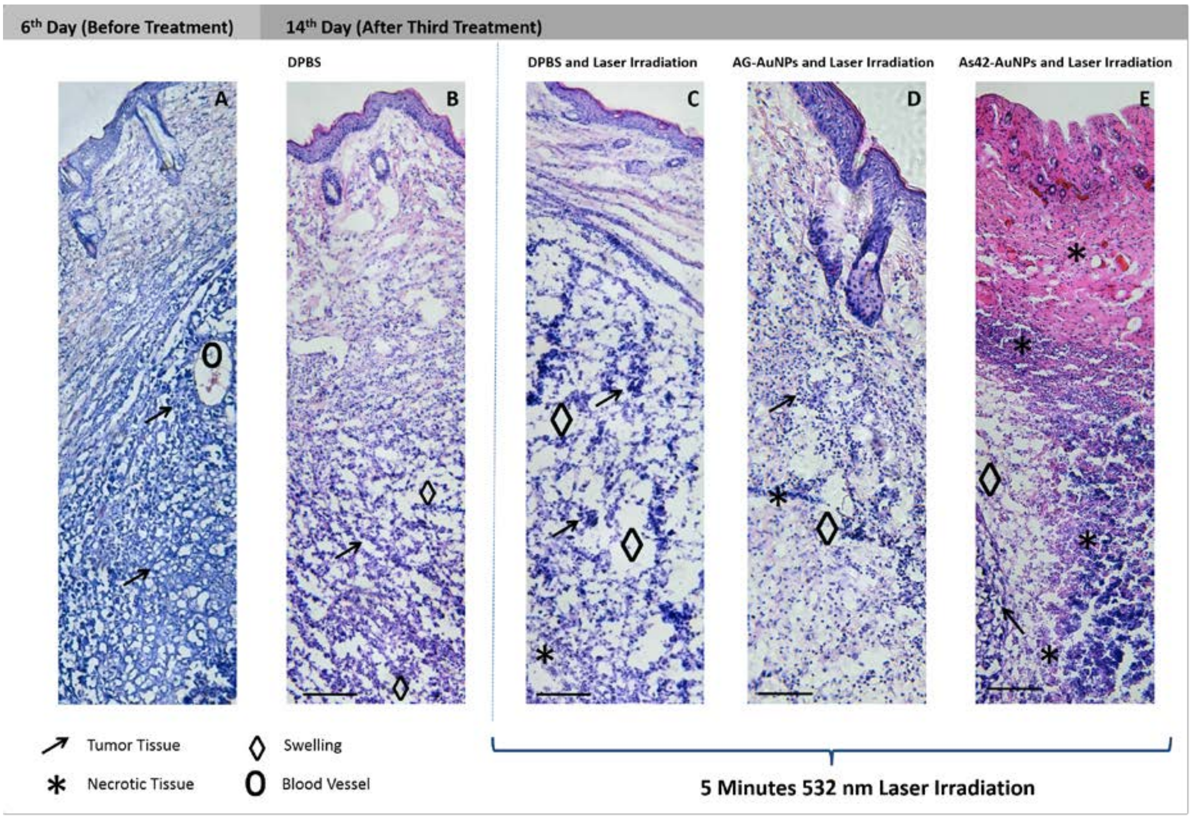

Figure 1S. General view of histopathological changes of solid Ehrlich carcinoma assessed by H&E staining before PPT treatment (A), after tail vein injection of DPBS (B, C), AG-AuNPs (D), AS42-AuNPs (E) after 5 min of laser irradiation at 1.2 Watts. Magnification×50.
